# Flourishing as an Aim of Medical Education: Are We Hitting the Target?

**DOI:** 10.1007/s40670-024-02255-x

**Published:** 2025-01-29

**Authors:** Noreen Mansuri, Amy B. Zelenski

**Affiliations:** 1 School of Medicine, University of California, Davis, Sacramento, CA USA; 2 School of Medicine and Public Health, University of Wisconsin, Madison, WI USA

**Keywords:** Medical education, Aims, Flourishing, Wellbeing, Medical students

## Abstract

In this commentary, we use Reiss and White’s contention of educational aims as a lens to examine the aims of medical education and determine whether the flourishing of medical students is among them. We identify an absence of flourishing and observe descriptions of medical students as finished products of training with an emphasis on professional virtues such as altruism. This emphasis is a compensatory response to professional and cultural shifts during the twentieth century. Anchored by this historical context, we draw on the work of Fielding and Moss to offer a path forward for redefining the aims of medical education.

## Introduction

Since medical student mistreatment was publicly recognized in 1982, attention to trainee wellness and wellbeing has steadily increased [1]. However, there is a burgeoning backlash to this focus on trainee, particularly medical student and junior resident, wellness. In a recent publication, Dr. Lisa Rosenbaum contends that the preoccupation with wellness has created an aversion to the inevitable hardships and sacrifices of medical training [2]. Though Dr. Rosenbaum argues for the need to help trainees better distinguish between genuine harm and the challenges necessary for their growth and skill advancement, there is an underlying assumption that a focus on student wellbeing is a threat to clinical excellence. Treating wellbeing and excellence as a binary opposition will limit our ability to address current problematic trends.

Despite the increased attention on wellness, nearly 30% of medical students are depressed and 11% endorse suicidal ideation [3]. Almost half of all students experience burnout [4], a state of emotional exhaustion that is associated with increased medical errors [5] and decreased empathy [6]. For medical students, the decline in empathy corresponds with their third, and first fully clinical, year of training [7, 8]. These trends are all the more concerning when we consider that students enter medical school with better mental health indicators than their peers [9] and a high degree of humanism [10], and that they continue to experience depressive symptoms and suicidal ideations in residency and attendinghood at higher rates than the general population [11–13]. In response to these trends, medical school leadership have incorporated positive changes such as duty hour limitations, pass/fail grading systems, and wellness programming such as self-care, meditation and resiliency training [14]. However, medical educators and students alike have critiqued these programs, noting that they often reflect popular trends in the wellbeing industry rather than meeting students’ needs [15, 16]. Thus, the persistence of such data reveals the irony of our profession’s pledge to ‘do no harm’ to patients while simultaneously harming trainees. Unsettled by this irony, we wondered: can medical training attend to the health of *both* patients and students? Combining our perspectives as a medical student and medical educator, we find it productive to frame and engage with this question as a reflection on the aims of medical education. We were guided to this reflective path by education scholars Reiss and White [17] and Fielding and Moss [18].

Reiss and White contend that the fundamental aims of education are to cultivate in students the skills they need to flourish individually and support the flourishing of others [17]. Though developed in the context of school education in the UK, these aims are remarkably relevant to medical training. Sharing a similar perspective to Reiss and White, Fielding and Moss provide educators with a list of questions for critically reflecting upon and thoughtfully developing educational aims [18]. Given the history of exchange between medicine and other education fields [19], these arguments are a prompt for medical educators to reflect on the aims of medical education, particularly whether the care of medical students is among them.

To meet this call, we examined the stated aims of medical education. In this commentary, we will elaborate on why such an examination is warranted. After doing so, we will describe the aims of medical education published in books and journals, and by national governing bodies like the Association of American Medical Colleges (AAMC) and Liaison Committee on Medical Education (LCME) over the last 30 years. We will contextualize these aims within several professional shifts that occurred during the twentieth century. This historical analysis will serve as a springboard to how Reiss and White’s proposed educational aims are a viable framework for medical educators to meet the current needs of medical education. We will conclude with an offering for how medical educators can continue contemplating and redefining educational aims at their institutions using the work of Fielding and Moss.

## The State of Medical Education

Medical school is mentally, emotionally and physically demanding. During their preclinical years, medical students encounter a large volume of information at such a fast pace that both trainees and faculty liken it to ‘drinking from a firehose’. Medical students must quickly master this information in preparation for clinical rotations and to demonstrate their knowledge on national licensing examinations prior to graduating with their degree. Their performances during clinical rotations and on national board exams are critical components of their residency applications. Since medical students are not guaranteed a residency spot, even though it is required for medical licensing, becoming a competitive residency applicant through high scores, extensive extra-curricular activities, and publications is a priority for students from the start of medical school. Though this high-pressure education system has created a ceaseless horse-race to and throughout medical school, forcing students to become performance-focused, they are criticised by some educators for exhibiting a “near total loss” in the love for learning [20].

In addition to the academic demands of medical school and the competition for residencies, students must also navigate a hierarchical professional culture, one of the defining features of medicine’s professional hierarchy hidden curriculum [21]. Research has shown that within medicine’s professional hierarchy, 64–76% of medical students report at least one episode of mistreatment by faculty and residents, with 11–13% experiencing recurrent mistreatment [1]. Mistreatment manifests in practices like ‘pimping’, where senior team members publicly assess junior team members’ knowledge in ways that intimidate or humiliate them and reinforce the team hierarchy [22, 23], and as microaggressions toward BIPOC students who are underrepresented in medicine (URiM) [24]. Microaggressions have insidious consequences and can promote feelings of shame and imposterism in students [25], as well as compromise their psychological [26] and identity safety [27]. Recent research in other STEM fields suggest that institutional and structural racism have been incorrectly chalked up to imposter syndrome which, in turn, inappropriately holds URiM students responsible for the entrenched environmental and cultural factors acting up on them [28]. These academic and professional stresses are further exacerbated by the everyday emotional and moral challenges of patient care which cause the “traumatic de-idealization” of medical practice in students [8]. Unsurprisingly, studies show that the pressure and culture of medical training adversely impact trainees [15]. Are these adverse effects the intended aims of medical education? Certainly not. So, what are? In the following section, we examine some of the articulated aims over the last 30 years.

## Current Aims of Medical Education

In *Understanding Medical Education: Evidence, Theory and Practice*, Swanwick states that the ‘ultimate’ aim of medical education is “to supply society with a knowledgeable, skilled and up-to-date cadre of healthcare professionals who put patient care above self-interest, and who undertake to maintain and develop their expertise over the course of a lifelong career” [29]. Similarly, Pugsley and McCrorie advise that medical education must meet the public’s expectation to “produce safe, ethical, and professional doctors” [30]. In these descriptions, medical students are not mentioned, but referenced as finished products (e.g., “health professionals” and “professional doctors”). Instead, society and professional virtues are centred.

The emphasis placed on professionalism is in response to the public’s dwindling trust in the medical profession [10, 31]. In the USA, this distrust unfolded throughout the twentieth century. In the early 1900s, American medicine witnessed a rise in for-profit medical schools that were graduating poorly trained physicians [31]. To address this problem, the American Medical Association (AMA) commissioned a survey of medical schools to provide recommendations for standardizing and improving medical education [32]. This investigation was headed by Abraham Flexner who argued that medicine is a principally scientific endeavour and requires university-based training in laboratory research [31]. Flexner’s perspective was readily adopted and used to structure US medical education in a way that privileged the basic sciences over clinical skills and bedside teaching which had literally and figuratively placed patients at the centre of care [31]. In addition to transforming medical school curriculum and pedagogy, Flexner’s restructuring of medical education had profound socio-cultural impacts on the profession. Most significantly, Flexner’s report led to the closure of five of the seven extant Black medical schools at the time. Their closure led to the devastating loss of an estimated 35, 315 Black physicians from 1910 to 2019 [33].

While Flexner’s overhaul of medical education occurred in the first half of the twentieth century, the second half was shaped by the increasing commercialization of healthcare [10, 34]. Commercialization led to public concerns over physician’s self-interest in clinical practice which only exacerbated patients’ dissatisfaction with the detached, cerebral approach to medicine that dominated in the aftermath of the Flexner report [31]. Worse, still, were the cases of reprehensible human experimentation like the Tuskegee Syphilis Study—where the cure for Syphilis (Penicillin) was intentionally withheld from affected Black men [35]—that further called into question the trustworthiness of doctors. Recognizing the increasingly negative reputation of the medical profession, its leaders sought to restore professionalism in their social contract with the public [10].

In 1996, the Association of American Medical Colleges (AAMC) commissioned the Medical School Objectives Project (MSOP) to agree upon “the attributes that physicians need to meet society’s expectations of them” [36]. They concluded that physicians must be “altruistic”, “knowledgeable”, “skillful”, and “dutiful” [36]. Concomitantly, the American Board of Internal Medicine (ABIM) formed a subcommittee named Project Professionalism which emphasized “altruism as the essence of professionalism” [37]. These committees spurred the inclusion of professionalism coursework in medical education. However, educators observed that students did not respond positively to these curricular efforts because they often taught professionalism as a superficial list of dos and don’ts devoid of a deeper, reflective and ethical consciousness [38]. While these curricular attempts rightly sought to restore the primacy of professionalism to medical training, prescriptive lists lack meaning and do not prepare students for the public relations task ahead of them as physicians. We know that students’ mental health declines throughout medical school as they experience depression, isolation, mistreatment and burnout which are counterproductive to the provision of high-quality, empathic care [5, 38]. Harming trainees and then expecting of them altruism is incongruous and unsustainable. To address this incongruity, we should prioritize and articulate positive outcomes for medical students when delineating our aims for medical education. Looking to our colleagues in other education fields, Reiss and White provide us with a framework for attending to both patients and students simultaneously.

## Looking for Inspiration: Reiss and White’s Take on the Aims of Education

In their 2014 article, Reiss and White task themselves with defining the fundamental aims of school education [17]. Their focus on aims emerges from the observation that, in their UK context, school curriculum is developed by first considering the list of taught subjects and their requirements prior to articulating the overarching aims of the composite curriculum. In this chronology, aims are an afterthought, “tacked on” with little effect on curriculum content [17]. Therefore, Reiss and White urge school educators to consider the aims *first*, resonant with the Backward Design model in K-12 education that has been increasingly adopted by medical education in the transition to competency-based curricula [39].

To their credit, Reiss and White recognize this is not an easy undertaking given the multifarious perspectives on the aims of school education. After surveying and analyzing the writings of numerous education scholars, they propose “two fundamental aims of school education, namely, to enable each learner to lead a life that is personally flourishing and to help others to do so too” [17]. One might ask, what do they mean by “flourishing”?

Reiss and White describe flourishing as a “life of autonomous, whole-hearted and successful engagement in worthwhile relationships, activities, and experiences” [17]. Their definition resembles those developed in positive psychology where flourishing is thought of “as experiencing five pillars of well-being that collectively result in feeling good and living well: positive emotions, engagement, positive relationships, meaning, and accomplishments” [40]. As indicated by this definition, flourishing accounts for multiple dimensions of wellbeing. In medicine, these dimensions have not always been acknowledged. Because of its more robust conceptualisation, flourishing has received attention in both clinical practice [41, 42] and medical education [43].

In 2011, Slavin et al. advocated for the need to promote medical student and resident flourishing, defined as “an individual state of well-being, characterized by positive emotion, engagement, strong relationships, meaning, and achievement” [43]. They argue that there is a lack of student flourishing given the immense cognitive load, prevalence of burnout and limited time with loved ones during training. To promote medical student flourishing, they recommend medical schools institute mindfulness and resiliency trainings, promote engagement through extra-curricular opportunities, invest in student bonding activities outside the classroom and celebrate non-academic accomplishments. More recently, Slavin has argued for shifting the focus to increasing trainee satisfaction—a positive emotion—with school, self, and life in general [16]. While these suggestions are valuable and capture several of the flourishing criteria, they remain superficial to the underlying problem: medical student flourishing is not a central aim or mission of medical education.

This absence is evident when examining the guidelines of the Liaison Committee for Medical Education (LCME), jointly led by the AAMC and AMA, which is responsible for accrediting medical schools [44]. In their updated guidelines, the LCME delineates 12 standards that medical schools must meet to receive or maintain accreditation. Included in the 12th standard, “Medical Student Mental Health Services, Personal Counseling, and Financial Aid Services”, the LCME expects that:

“A medical school has in place an effective system of counseling services for its medical students that includes programs to promote their well-being and to facilitate their adjustment to the physical and emotional demands of medical education” [45].

In the appendix, the LCME defines a wellbeing program as “an organized and coordinated program designed to maintain or improve physical, emotional and mental health through proper diet, exercise, stress management and illness prevention” [45]. Not only is this guidance vague, but it employs a narrow view of wellbeing that relies on the same wellness industry trends (e.g., “diet”, “exercise” and “stress management”) that medical students have critiqued for not meeting their actual needs [15, 16], fueling the impression that they are ‘tacked on’. Furthermore, the “adjustment” called for does not distinguish between preparing students for a rigorous medical education that is in service of a successful professional career from antiquated cultural norms in need of change. The former is more in line with flourishing and focuses on equipping students with knowledge and skills (i.e. agency) for excelling in their training environment rather than “adjustment” which is passive and defeatist in nature.

Given the vagueness of the above guidance, medical schools have substantial autonomy in developing their wellness objectives and programming. There are individual schools that are attending to this. We attempted to access information about individual allopathic institutions’ aims through their websites, published strategic plans for program improvement, and the Medical Student Admission Requirements (MSAR) Report [46], but determined them too crude a measure to ‘know’ how these schools are fostering flourishing (or not). As we strive to integrate flourishing into intuitional aims and programming, it is important to evaluate their impact, and there are many ways to do so. For example, Kelly-Hedrick et al. use a combination of validated measures and questionnaires, while Whitaker et al. employ other, non-traditional qualitative methods such as observation, all of which can be utilized for quality improvement and program accountability [47, 48].

We note that, by discussing the importance of protecting and preserving student wellbeing, we are not challenging the centrality of patients in the aims of medical education; rather, we argue that the flourishing of both students and patients should be linked for theirs is a symbiotic relationship. Though they are not the focus of this perspective piece, we whole-heartedly acknowledge that flourishing is also essential for practicing clinicians who lead medical training and that there are innumerable individual educators who are deeply committed to the flourishing of medical students. As Dr. Robin Wall Kimmerer asserts, “all flourishing is mutual” [49]. We must create a system that holds this idea at the centre of medical education. This is precisely what Reiss and White remind us of.

## Moving Forward: Asking the Right Questions with Fielding and Moss

While writing this perspective, we have been compelled to ask ourselves: *To what extent are medical students valued? Who is responsible for preserving medical students’ mental health and humanistic instinct?* While these questions are challenging to answer, they should not be avoided. In their book, *Radical Education and the Common School*, Fielding and Moss make the case for why it is essential to critically interrogate education, beginning with its aims and values. When we do not, education becomes an instrument of the status quo and ceases to serve students, educators and the public [18]. In the case of medical education, where service to the public is a priority, we cannot afford to be complacent. To prevent complacency, Fielding and Moss encourage educators to ask a list of ten critical questions [18], which we have adapted for medical education in Table 1.

**Table 1:**
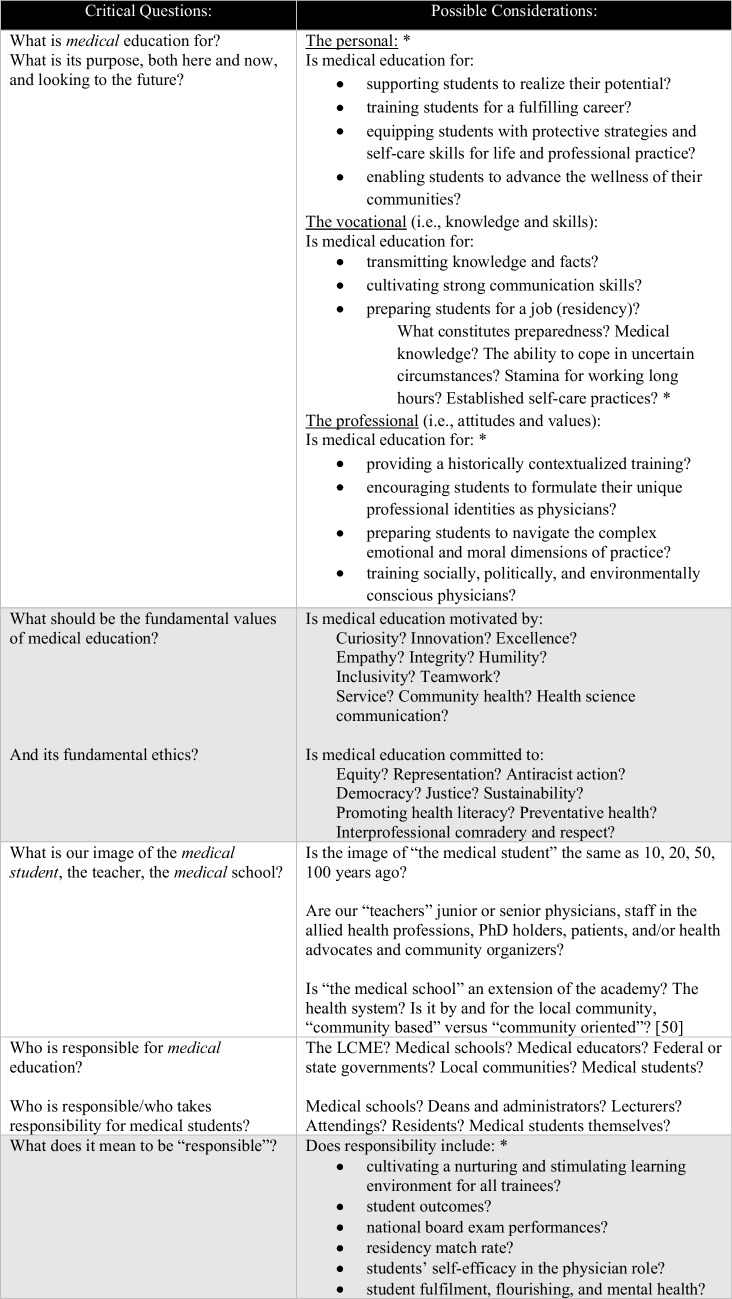
Critical questions adapted from Fielding and Moss to medical education [18]. The list of considerations in the adjoining column is neither exhaustive nor prescriptive, rather intended to guide and inspire reflection. Content demarcated with asterisks (*) highlight considerations related to of flourishing

In light of medical schools’ autonomy over their individual educational objectives, they are poised to ask these questions. We all can and should do this as a way to catalyze meaningful action that is capable of addressing the enduring and concerning trends involving trainee wellbeing. It is essential that this action involves students and is thoughtfully developed and longitudinal. After all, flourishing is not a box to check; it is a long-term effort undertaken for the good of students and patients.

## Conclusion

Professor Rita Charon has said that a physician’s best therapeutic agent is the self [51]. Unfortunately, however, the self is harmed throughout medical training. Instead of being prioritized as the recipients of medical education, medical students are regarded as products for public consumption. Medical schools would better serve the public by attending to the mutual flourishing of patients and trainees in the aims of medical education. Valuing students in such a way would not detract from the rigor of training; it *would* better sustain them for decades-long clinical careers that lead to both personal fulfillment and high-quality patient care.

## References

[1] Cook AF, Arora VM, Rasinski KA, Curlin FA, Yoon JD. The prevalence of medical student mistreatment and its association with burnout. Acad Med J Assoc Am Med Coll. 2014;89(5):749–54. 10.1097/ACM.0000000000000204. 10.1097/ACM.0000000000000204 PMC4401419 24667503

[2] Rosenbaum L. Being well while doing well - distinguishing necessary from unnecessary discomfort in training. N Engl J Med. 2024;390(6):568–72. 10.1056/NEJMms2308228. 38231543 10.1056/NEJMms2308228

[3] Rotenstein LS, Ramos MA, Torre M, Segal JB, Peluso MJ, Guille C, et al. Prevalence of depression, depressive symptoms, and suicidal ideation among medical students: a systematic review and meta-analysis. JAMA. 2016;316(21):2214–36. 10.1001/jama.2016.17324. 27923088 10.1001/jama.2016.17324 PMC5613659

[4] Frajerman A, Morvan Y, Krebs MO, Gorwood P, Chaumette B. Burnout in medical students before residency: a systematic review and meta-analysis. Eur Psychiatry. 2019;1(55):36–42. 10.1016/j.eurpsy.2018.08.006. 10.1016/j.eurpsy.2018.08.006 30384110

[5] West CP, Huschka MM, Novotny PJ, Sloan JA, Kolars JC, Habermann TM, et al. Association of perceived medical errors with resident distress and empathy: a prospective longitudinal study. JAMA. 2006;296(9):1071–8. 10.1001/jama.296.9.1071. 16954486 10.1001/jama.296.9.1071

[6] Thomas MR, Dyrbye LN, Huntington JL, Lawson KL, Novotny PJ, Sloan JA, et al. How do distress and well-being relate to medical student empathy? A Multicenter Study. J Gen Intern Med. 2007;22(2):177–83. 10.1007/s11606-006-0039-6. 17356983 10.1007/s11606-006-0039-6 PMC1824738

[7] Hojat M, Vergare MJ, Maxwell K, Brainard G, Herrine SK, Isenberg GA, et al. The Devil is in the third year: a longitudinal study of erosion of empathy in medical school. Acad Med. 2009;84(9):1182. 10.1097/ACM.0b013e3181b17e55. 19707055 10.1097/ACM.0b013e3181b17e55

[8] Chen DCR, Kirshenbaum DS, Yan J, Kirshenbaum E, Aseltine RH. Characterizing changes in student empathy throughout medical school. Med Teach. 2012;34(4):305–11. 10.3109/0142159X.2012.644600. 22455699 10.3109/0142159X.2012.644600

[9] Brazeau CMLR, Shanafelt T, Durning SJ, Massie FS, Eacker A, Moutier C, et al. Distress among matriculating medical students relative to the general population. Acad Med. 2014;89(11):1520. 10.1097/ACM.0000000000000482. 25250752 10.1097/ACM.0000000000000482

[10] Hilton S, Southgate L. Professionalism in medical education. Teach Teach Educ. 2007;23(3):265–79. 10.1016/j.tate.2006.12.024.

[11] Mata DA, Ramos MA, Bansal N, Khan R, Guille C, Di Angelantonio E, et al. Prevalence of depression and depressive symptoms among resident physicians: a systematic review and meta-analysis. JAMA. 2015;314(22):2373. 10.1001/jama.2015.15845. 26647259 10.1001/jama.2015.15845 PMC4866499

[12] Kalmoe MC, Chapman MB, Gold JA, Giedinghagen AM. Physician Suicide: A Call to Action. Mo Med. 2019;116(3):211–6. 31527944 PMC6690303

[13] Shanafelt TD, Dyrbye LN, West CP, Sinsky C, Tutty M, Carlasare LE, et al. Suicidal ideation and attitudes regarding help seeking in US physicians relative to the US working population. Mayo Clin Proc. 2021;96(8):2067–80. 10.1016/j.mayocp.2021.01.033. 34301399 10.1016/j.mayocp.2021.01.033

[14] Schutt A, Chretien KC, Woodruff JN, Press VG, Vela M, Lee WW. National survey of wellness programs in U.S. and Canadian medical schools. Acad Med. 2021;96(5):728. 10.1097/ACM.0000000000003953. 33538474 10.1097/ACM.0000000000003953

[15] Chatterjee K, Edmonds VS, Girardo ME, Vickers KS, Hathaway JC, Stonnington CM. Medical students describe their wellness and how to preserve it. BMC Med Educ. 2022;22(1):1–11. 10.1186/s12909-022-03552-y. 35764972 10.1186/s12909-022-03552-y PMC9241274

[16] Slavin S. Reimagining well-being initiatives in medical education: shifting from promoting wellness to increasing satisfaction. Acad Med. 2021;96(5):632. 10.1097/ACM.0000000000004023. 33635840 10.1097/ACM.0000000000004023

[17] Reiss MJ, White J. An aims-based curriculum illustrated by the teaching of science in schools. Curric J. 2014;25(1):76–89. 10.1080/09585176.2013.874953.

[18] Fielding M, Moss P. The state we’re in. In: Radical Education and the Common School. Routledge; 2010. pp. 1–38. 10.4324/9780203837405

[19] McKnight L, Morgan A. A broken paradigm? What education needs to learn from evidence-based medicine. J Educ Policy. 2020;35(5):648–64. 10.1080/02680939.2019.1578902.

[20] Tough Love: NOS Episode 2.3. N Engl J Med [Internet]. 2024 Feb 15 [cited 2024 May 23];390(7). Available from10.1056/NEJMp2400690. 10.1056/NEJMp2400690 38354137

[21] Gaufberg EH, Batalden M, Sands R, Bell SK. The hidden curriculum: what can we learn from third-year medical student narrative reflections? Acad Med J Assoc Am Med Coll. 2010;85(11):1709–16. 10.1097/ACM.0b013e3181f57899. 10.1097/ACM.0b013e3181f57899 20881818

[22] Kherani IZ, Sharma M. Toward trauma-informed pedagogy: an intersectional analysis of pimping in medical education. Acad Med. 2022;97(9):1295. 10.1097/ACM.0000000000004724. 35507457 10.1097/ACM.0000000000004724

[23] Kost A, Chen FM. Socrates was not a pimp: changing the paradigm of questioning in medical education. Acad Med J Assoc Am Med Coll. 2015;90(1):20–4. 10.1097/ACM.0000000000000446. 10.1097/ACM.0000000000000446 25099239

[24] Chisholm LP, Jackson KR, Davidson HA, Churchwell AL, Fleming AE, Drolet BC. Evaluation of racial microaggressions experienced during medical school training and the effect on medical student education and burnout: a validation study. J Natl Med Assoc. 2021;113(3):310–4. 10.1016/j.jnma.2020.11.009. 33358632 10.1016/j.jnma.2020.11.009

[25] Bynum WE, Varpio L, Lagoo J, Teunissen PW. “I’m unworthy of being in this space”: the origins of shame in medical students. Med Educ. 2021;55(2):185–97. 10.1111/medu.14354. 32790934 10.1111/medu.14354

[26] McClintock AH, Fainstad T. Growth, engagement, and belonging in the clinical learning environment: the role of psychological safety and the work ahead. J Gen Intern Med. 2022;37(9):2291–6. 10.1007/s11606-022-07493-6. 35710656 10.1007/s11606-022-07493-6 PMC9296742

[27] Bullock JL, Sukhera J, Del Pino‐Jones A, Dyster TG, Ilgen JS, Lockspeiser TM, et al. ‘Yourself in all your forms’: a grounded theory exploration of identity safety in medical students. Med Educ. 2023;medu.15174. 10.1111/medu.15174. 10.1111/medu.15174 37517809

[28] McGee EO, Botchway PK, Naphan-Kingery DE, Brockman AJ, Houston S, White DT. Racism camouflaged as impostorism and the impact on Black STEM doctoral students. Race Ethn Educ. 2022;25(4):487–507. 10.1080/13613324.2021.1924137.

[29] Swanwick T. Understanding medical education. In: Understanding Medical Education. John Wiley & Sons, Ltd; 2018; pp 1–6. 10.1002/9781119373780.ch1

[30] Pugsley L, McCrorie P. Improving medical education: improving patient care. Teach Teach Educ. 2007;23(3):314–22. 10.1016/j.tate.2006.12.023.

[31] Duffy TP. The Flexner Report - 100 years later. Yale J Biol Med. 2011;84(3):269–76. 21966046 PMC3178858

[32] Starr P. The social transformation of American medicine. Updated. New York: Basic Books; 2017.

[33] Campbell KM, Corral I, Infante Linares JL, Tumin D. Projected estimates of African American medical graduates of closed historically Black medical schools. JAMA Netw Open. 2020;3(8):e2015220. 10.1001/jamanetworkopen.2020.15220. 32816033 10.1001/jamanetworkopen.2020.15220 PMC7441360

[34] Irby DM, Cooke M, O’Brien BC. Calls for reform of medical education by the Carnegie Foundation for the Advancement of Teaching: 1910 and 2010. Acad Med J Assoc Am Med Coll. 2010;85(2):220–7. 10.1097/ACM.0b013e3181c88449. 10.1097/ACM.0b013e3181c88449 20107346

[35] Wailoo K. Patients are humans too: the emergence of medical humanities. Daedalus. 2022;151(3):194–205. 10.1162/daed_a_01938.

[36] The Medical School Objectives Writing Group. Learning objectives for medical student education— guidelines for medical schools: report I of the Medical School Objectives Project [Internet]. Association of Americal Medical Colleges; 1998; pp 1–16. Available from: https://store.aamc.org/downloadable/download/sample/sample_id/144/. Accessed 4 Dec 2024. 10.1097/00001888-199901000-00010 9934288

[37] American Board of Internal Medicine. Project Professionalism [Internet]. 2001; pp 1–46. Available from: https://medicinainternaucv.wordpress.com/wp-content/uploads/2013/02/project-professionalism.pdf. Accessed 4 Dec 2024.

[38] Brody H, Doukas D. Professionalism: a framework to guide medical education. Med Educ. 2014;48(10):980–7. 10.1111/medu.12520. 25200018 10.1111/medu.12520

[39] Joyce BL, Swanberg SM. Using backward design for competency-based undergraduate medical education. In: Stefaniak J, editor. Advancing Medical Education Through Strategic Instructional Design [Internet]. Hershey: IGI Global; 2017. p 53-76. Available from 10.4018/978-1-5225-2098-6.ch003.

[40] Cherkowski S, Kutsyuruba B, Walker K. Positive leadership: animating purpose, presence, passion and play for flourishing in schools. J Educ Adm. 2020;58(4):401–15. 10.1108/JEA-04-2019-0076.

[41] VanderWeele TJ. On the promotion of human flourishing. Proc Natl Acad Sci. 2017;114(31):8148–56. 10.1073/pnas.1702996114. 28705870 10.1073/pnas.1702996114 PMC5547610

[42] VanderWeele TJ, McNeely E, Koh HK. Reimagining health—flourishing. JAMA. 2019;321(17):1667–8. 10.1001/jama.2019.3035. 30933213 10.1001/jama.2019.3035

[43] Slavin SJ, Hatchett L, Chibnall JT, Schindler D, Fendell G. Helping medical students and residents flourish: a path to transform medical education. Acad Med J Assoc Am Med Coll. 2011;86(11):e15. 10.1097/ACM.0b013e3182316558. 10.1097/ACM.0b013e3182316558 22030663

[44] Kassebaum DG. Origin of the LCME, the AAMC-AMA partnership for accreditation. Acad Med J Assoc Am Med Coll. 1992;67(2):85–7. 10.1097/00001888-199202000-00005. 10.1097/00001888-199202000-00005 1547000

[45] Liaison Committee on Medical Education (LCME). Standards, Publications, & Notification Forms | LCME [Internet]. 2024. Available from: https://lcme.org/publications/. Accessed 4 Dec 2024.

[46] Association of American Medical Colleges. Medical School Admission Requirements (MSAR) Report for Applicants and Advisors--Mission Statement 2025 [Internet]. 2024. Available from: https://students-residents.aamc.org/media/6966/download?attachment. Accessed 4 Dec 2024.

[47] Kelly-Hedrick M, Iuliano K, Tackett S, Chisolm MS. Medical student flourishing before and during the COVID-19 pandemic at one U.S. institution. MedEdPublish. 2023;12:28. 10.12688/mep.19094.2. 36974117 10.12688/mep.19094.2 PMC10039319

[48] Whitaker RC, Payne GB, O’Neill MA, Brennan MM, Herman AN, Dearth-Wesley T, et al. Trauma-informed undergraduate medical education: a pathway to flourishing with adversity by enhancing psychological safety. Perspect Med Educ. 2024;13(1). 10.5334/pme.1173 10.5334/pme.1173 PMC11166023 38863986

[49] Kimmerer RW. Braiding sweetgrass: indigenous wisdom, scientific knowledge and the teachings of plants. Minneapolis: Milkweed Editions; 2013.

[50] Hays R. Community-oriented medical education. Teach Teach Educ. 2007;23(3):286–93. 10.1016/j.tate.2006.12.018.

[51] Charon R. Narrative medicine: honoring the stories of illness. Oxford, New York: Oxford University Press; 2006.

